# A novel paradigm to study spatial memory skills in blind individuals through the auditory modality

**DOI:** 10.1038/s41598-018-31588-y

**Published:** 2018-09-06

**Authors:** Walter Setti, Luigi F. Cuturi, Elena Cocchi, Monica Gori

**Affiliations:** 1 Unit for Visually Impaired People (U-VIP), Istituto Italiano di Tecnologia, Genoa, Italy; 2 Robotics, Brain and Cognitive Science (RBCS), Istituto Italiano di Tecnologia, Genoa, Italy; 3 David Chiossone Institute, Genoa, Italy; 4 DIBRIS Department, University of Genoa, Genoa, Italy

## Abstract

Spatial memory is a multimodal representation of the environment, which can be mediated by different sensory signals. Here we investigate how the auditory modality influences memorization, contributing to the mental representation of a scene. We designed an audio test inspired by a validated spatial memory test, the Corsi-Block test for blind individuals. The test was carried out in two different conditions, with non-semantic and semantic stimuli, presented in different sessions and displaced on an audio-tactile device. Furthermore, the semantic sounds were spatially displaced in order to reproduce an audio scene, explored by participants during the test. Thus, we verified if semantic rather than non-semantic sounds are better recalled and whether exposure to an auditory scene can enhance memorization skills. Our results show that sighted subjects performed better than blind participants after the exploration of the semantic scene. This suggests that blind participants focus on the perceived sound positions and do not use items’ locations learned during the exploration. We discuss these results in terms of the role of visual experience on spatial memorization skills and the ability to take advantage of semantic information stored in the memory.

## Introduction

In order to explore the surroundings of a familiar or unfamiliar environment, the brain needs to build a proper representation of the objects composing the surrounding space. These representations can be coded verbally, visually or with other sensory modalities. In many of these activities such as way-finding and objects localization, spatial memory plays a fundamental role. Scientific results suggest that the memorization of spatial contents works by storing information in two main formats: depictive (pictorial) and propositional (descriptive)^1^. In details, the first format refers to representations of objects that take into account specific spatial relationship between object’s parts and the second format indicates a verbal description of the object to be memorized. In the case of memorization of unfamiliar items, our brain mostly relies on propositional encoding of the information to be memorized^2^. Contrary, when faced with familiar items, our memorization is facilitated as we take advantage of both propositional and depictive strategies^3,4^. For instance, when we imagine an object or even a person, the stored representations of the object itself are activated in the high-level cortical areas and projected to perceptual visual areas through backward and forward connections^5^. In this context, imaginative strategies can be used to build mental images, defined as stable conscious representations of an object regardless of its complexity and in the absence of concomitant sensory stimuli^6^. According to the model proposed by Kosslyn^7^, the early visual areas such as V1 (so called “visual buffer” in the model) are activated during mental imagery. Since these cortical areas are topographically organized and depictive^1^, their activation supports the hypothesis that mental images involve depictive (or pictorial) representations.

In order to retrieve stored information and generate images from long-term memory (e.g. mental images), the brain must recruit working memory (WM). The WM system allows the simultaneous storage and processing of information^8^. According to the model proposed by Baddeley and Hitch^9^, WM is mainly divided into three subsystems: the central executive component (CE) that allows accessing information retained in the WM; the phonological loop, related to the verbal working memory (e.g. the retention of verbal information); the visuo-spatial working memory (VSWM), designed to retain and process visuo-spatial information. Recent work postulated the existence of a fourth component, namely the episodic buffer, that is a limited capacity system under the control of the CE^10^. Vecchi and co-workers^11^ modified the original structure of the model composed of independent subsystems, suggesting that these components are located along a continuum (“cone model”) characterized by two dimensions one of which is related to the level of control required by the WM process. This dimension is specifically suited to defining the relationship between imagery and the WM system and it indicates that in this process the subsystems are not completely independent but integrated. Mental imagery is usually seen as a product of VSWM or, according to other authors, of CE^12^. Considering the complexity of the processes underlying imagery abilities, Vecchi and co-workers hypothesize that peripheral subsystems and the central amodal components are not dissociated^11^.

An interesting research question regards what happens to the mental image generation when visual information is not available, as is the case of blindness. It is important to state that mental images are not just visual. For instance, congenitally blind people have knowledge about the world, derived through haptics^13^ or verbal instructions. It is well known that the representations generated on the basis of a previous haptic exploration, may be as accurate as images based on visual imagery^14^. Several works^14,15^ show that blind individuals are able to store and process mental images and representations of spatial information similarly to sighted participants by using the remaining sensory modalities to substitute vision in mental imagery processes. Collignon and colleagues^16,17^ showed that early visual deprivation can lead to functional cerebral cross-modal reorganization underlying auditory information processing. As a consequence, vision related cortical areas in blind individuals^18–21^ process non-visual sensory information encoded through the remaining senses. However, the ability to use mental images is slower in non-sighted individuals when compared to the sighted. Blind people indeed rely on non-visual spatial images whose elaboration processes require more time per se^22,23^. Supporting this view, Vecchi^24^ tested sighted and congenitally blind individuals, showing that the latter group is able to generate visuospatial images, but perform significantly worse than sighted participants in spatial memory tasks. These results indicate that vision seems to be the “preferred modality” for visuo-spatial working memory tasks and that spatial processing is strongly affected by the lack of vision. In addition, the performance of visually impaired individuals in a visuo-spatial memory task strongly depends on how large is the demand on memory. Visually impaired individuals show indeed poorer performance in tasks requiring high spatial memory load compared to the sighted individuals^25^. Furthermore, congenitally blind people have several difficulties whenever they are administered with a task that requires active processing abilities such as generating and manipulating several stimuli at a time^14,26^. On the contrary, both blind and sighted subjects have similar performance when accomplishing a passive task, during which they are asked to only retain information.

**Figure 1:**
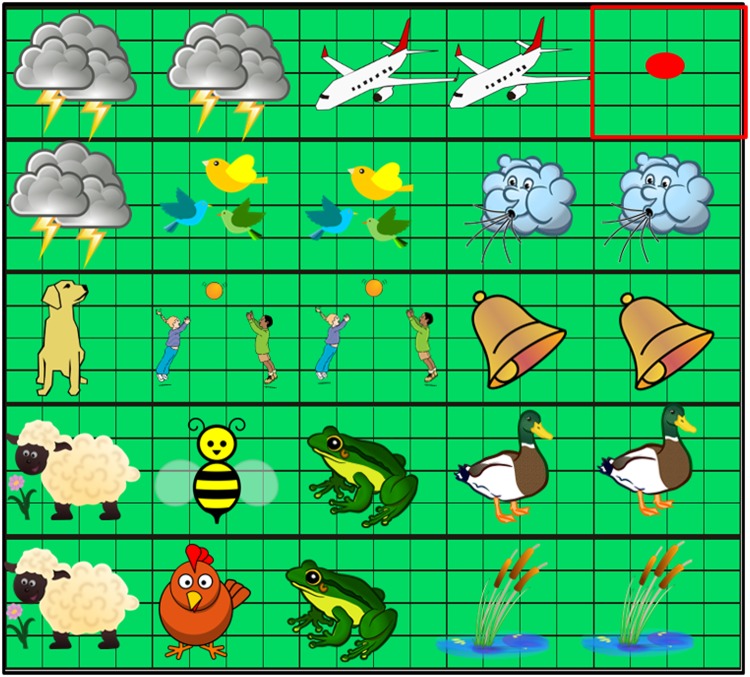
Audio scene. Each square represents a haptic block as indicated by the square highlighted in red on the top right corner of the figure (represented as plain for illustration purposes): the smaller squares composing the haptic block are the pads (16 for each block) and the speaker is at the centre of the haptic block (indicated by red dot). The sounds used in the semantic condition are represented as pictures displaced on the Arena surface, each of them indicates the sound the speaker emitted (e.g. bee buzzing or wind blowing). In order to make the scene realistic, sounds related to the sky are on the top of the device. Sounds related to the ground are at the centre, while sounds related to the nature (animals and pond) are emitted from the bottom loudspeakers. The haptic block on the top right corner emitted the airplane sound. The images showed in the figure were downloaded by a royalty-free images web archive (https://publicdomainvectors.org/).

The vast majority of studies on spatial imagery used touch and vision as primary sensory modalities and focused on simple images or structures^27^. Moreover, when focusing on visual imagery in blind individuals, previous works have not exploited the auditory modality (rather than touch)^11,23,24,28,29^ to convey complex images. Audition indeed allows to provide complex information, such as semantic or non-semantic content and its complexity can be varied by manipulating the presence of other sounds. Here we test for the first time how blind and sighted individuals process and memorize spatial complex auditory contents. First, we tested the role of visual experience on spatial audio memory. Second, we tested the role of visual experience derived by exploring a global audio scene on improving the processing of local audio signals. The aim of our work is to highlight the role of visual input in the development of spatial representation and to provide the basis to realize an audio memory test that can be used to compare spatial memory skills between blind and sighted individuals. To date, only a few paradigms are available to study spatial memory through the auditory channel and none, to our knowledge, has tested blind individuals with this sensory modality. We took inspiration from a well-known memory test, the “Corsi- Block”^30,31^ and we adapted it to the audio modality. In its original paradigm, the Corsi Block test consists of a set of 9 blocks displaced on a wooden board. The experimenter sequentially points to different blocks one after the other. After the tapping sequence is completed, the subject is asked to tap the blocks in the same order as done by the experimenter^32^. If a certain proportion of the sequence is correctly reproduced the sequence length is increased by one block. Over the years, the Corsi-Block test has frequently been used to assess visuospatial short-term memory performance in adults^33,34^, children^35^ and patients with neuropsychological deficits^36^. Differently from the original Corsi, we have used spatialized sounds instead of blocks. In addition, we have introduced an exploration phase during which participants have the possibility of developing their own mental image of the auditory scene through semantic sounds (Fig. 1). This paradigm was indeed divided into two conditions based on the stimuli we used: non-semantic and semantic. In the first condition, we used pure tones at different frequencies plus white noise. In the second condition, meaningful sounds (e.g. the thunderstorm, the bird calls). The latter were spatially arranged so that they would compose a coherent scene (e.g. sound of thunder coming from the upper space/loudspeakers, water from lower space/loudspeakers). The participants were tested in the two conditions separately. This paradigm allowed us to test whether a mental image of an auditory scene can enhance memorization processes. Both blind and sighted adults took part in the experiment. Our results show that the ability to recall sound sequences benefits more from the mental image of the audio scene in sighted individuals than in blind subjects.

## Results

**Figure 2:**
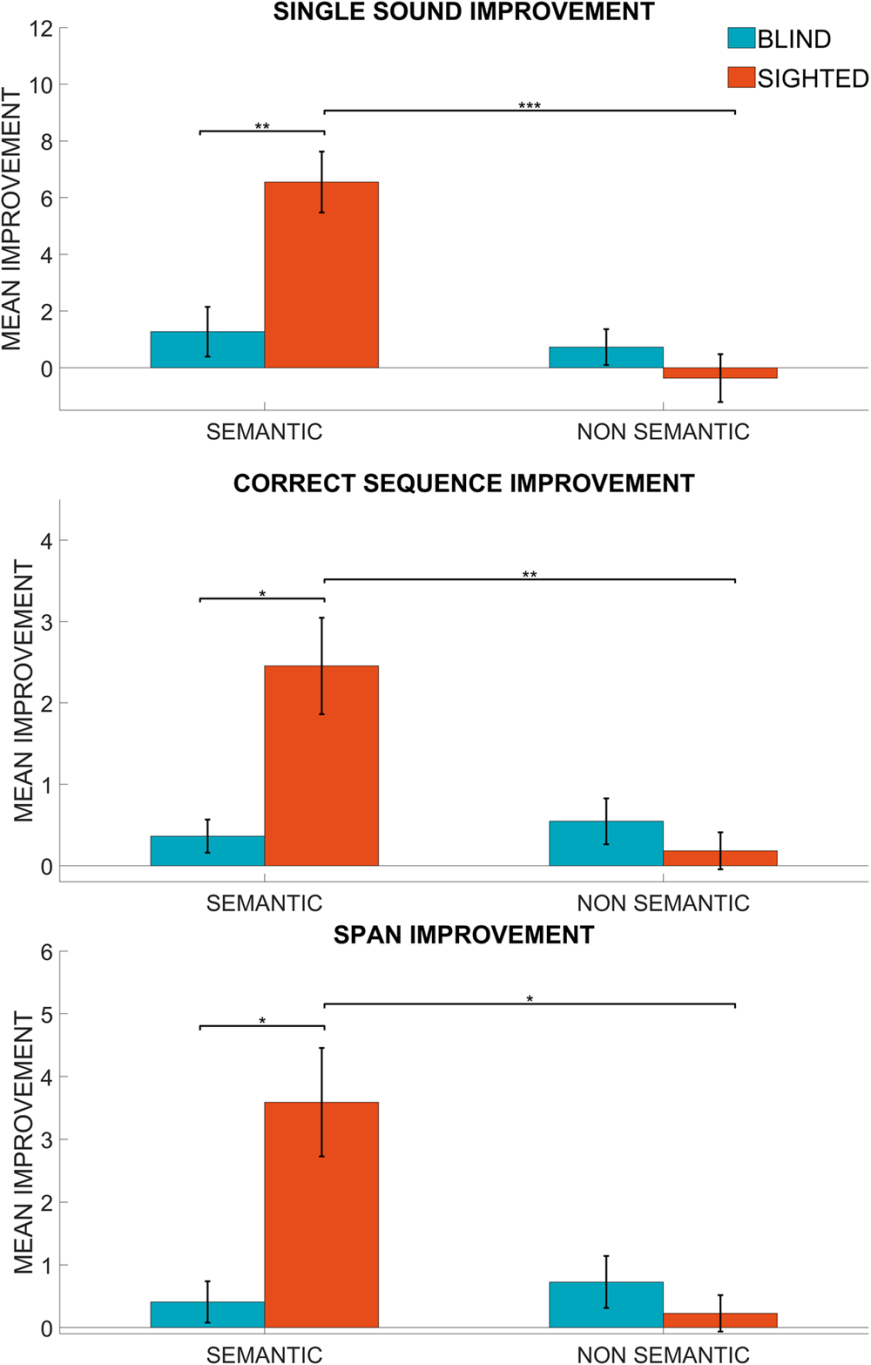
Improvement of the two groups in both conditions. Mean improvement indicates the difference between pre- and post-test in the number of single sounds correctly recalled (upper panel), the number of sequences correctly recalled (middle panel) and the span reached (lower panel). In all cases, semantic improvement of sighted participants is significantly greater than their non-semantic improvement and greater than semantic improvement in blind participants as indicated by the asterisks (one asterisk (*) represents p < 0.05, two asterisks (**) represent p < 001 and three asterisks (***) represent p < 0.001). There was no significant difference of improvement for blind participants between semantic and non-semantic conditions.

We tested both sighted and blind individuals. All groups performed the test twice, in two conditions: with semantic and with non-semantic sounds. Participants sat in front of an audio-tactile device, named Arena, at a fixed distance of 30 cm. Arena is a 5-by-5 matrix of speakers, covered by tactile sensors (Fig. 1). In the pre-test phase, participants listened to sequences of sounds coming from different loudspeakers. In the exploration phase, participants haptically explored the device while listening to the sounds produced at different spatial positions after each touch. In the last phase (post-test), we presented, as in the pre-test phase, a set of sound sequences. All participants were tested with the same sounds in both pre- and post- test phases but the sequences were varied. In both test phases the task was to reproduce the correct spatial sequences of the sounds presented. A single response was considered correct if the subject touched Arena at a distance less than 14 cm (distance between the centres of two consecutives speakers in the diagonal direction) from the position of the loudspeaker that emitted the sound. In the analysis, the performance was evaluated considering three parameters: the number of single sounds in the sequences correctly recalled out of the total (18 sounds) as a general measure; the number of sequences correctly recalled out of the total (6 sequences); and the span (defined as (1) in Material and Methods). Results were first analysed in terms of memory improvement, i.e. the difference in performance between the post- and the pre-test phases. Statistical analysis was carried out in Matlab (Matlab R2017a, The Mathworks) and the data are presented as mean and standard error. We ran 2-way (2 × 2) mixed measures model ANOVA, for each of the three parameters described above, with *group* (blind vs. sighted) as between factor and *condition* (semantic vs. non-semantic) as within factor. In Fig. 2, results are shown for the two groups. The analysis of variance revealed significant interactions between *group* and *condition* for single sound, (F(1,20) = 15.49, p < 0.001), span (F(1,20) = 14.45, p < 0.01) and correct sequence improvements (F(1,20) = 14.46, p < 0.01). Post-hoc analysis were carried out with two tailed Student’s test, both paired and unpaired. Bonferroni correction was used to correct for multiple comparisons (p < 0.05 was considered significant). The analysis of sighted participants performance, shows better results in the semantic compared to the non-semantic condition (paired t-test, single sound improvement: t = 3.83, p < 0.001, correct sequence improvement: t = 3.85, p < 0.05, span improvement: t = 5.39, p < 0.05). For the blind participants instead we did not find any significant difference between the conditions. In addition, sighted participants performed better in the semantic condition compared to blind subjects (unpaired t-test, single sound improvement: t = 3.8079, p < 0.01, correct sequence improvement: t = 3.33, p < 0.05, span improvement: t = 3.71, p < 0.05). This suggests that sighted participants benefit more from exploration than blind subjects.

**Figure 3:**
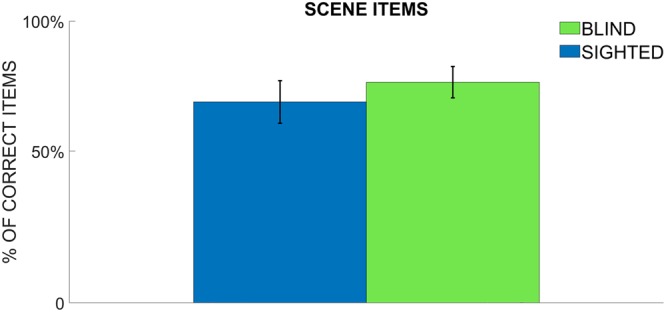
Mean percentage of audio items recalled out of the total. The bar plots indicate the mean percentage of audio items which participants were able to correctly recall at the end of the semantic condition.

In order to verify whether this outcome was due to the fact that sighted participants remembered a higher number of items’ positions rather than blind subjects, at the end of the semantic post-test participants were asked to indicate the position of each element of the scene. This is illustrated in Fig. 3 where we represent the number of recalled items’ positions for the two groups. Post-hoc analysis revealed no significant difference between sighted and blind subjects indicating that they are equally able to recall the positions of single audio items displaced on Arena.

**Figure 4:**
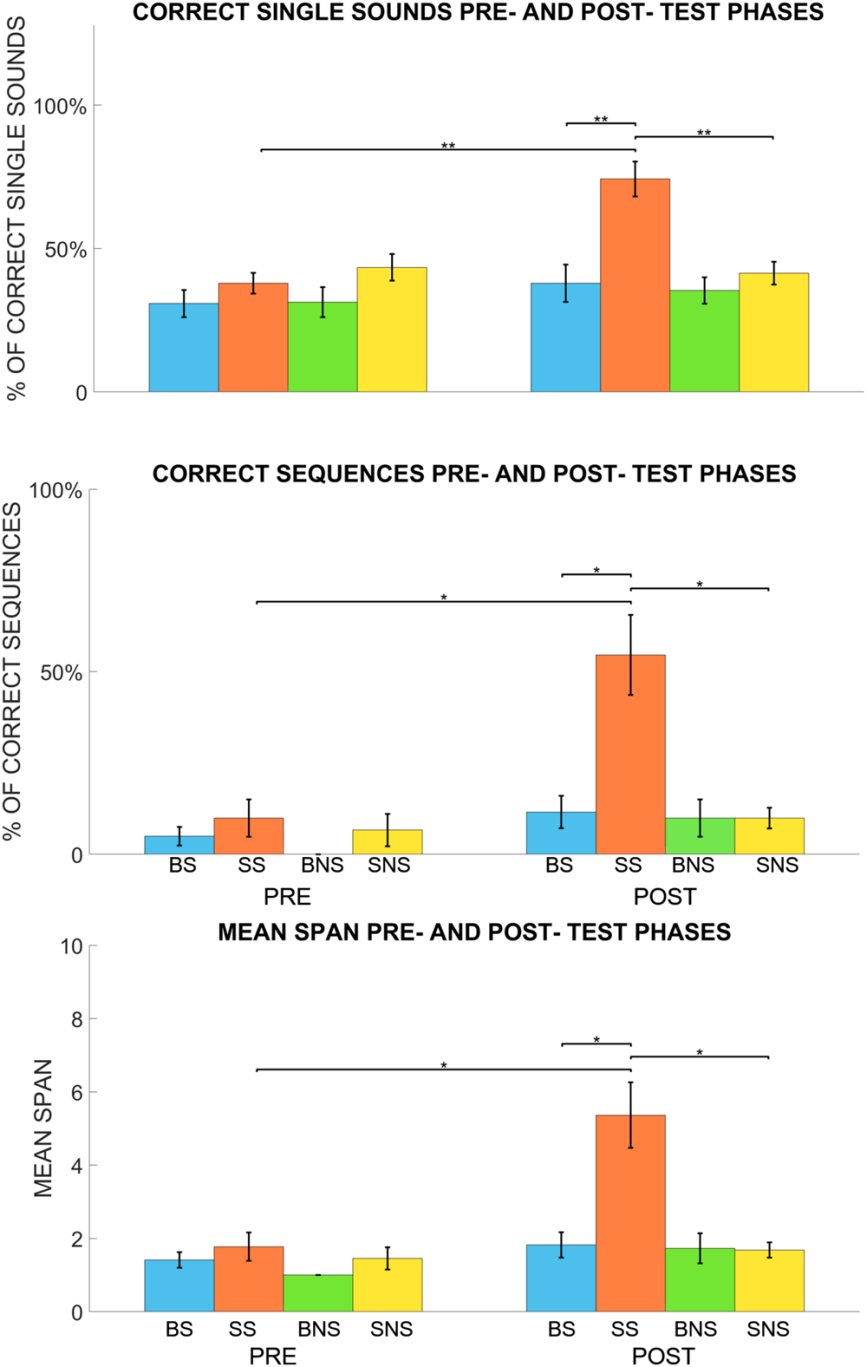
Comparisons between pre- and post- test phases. BS, BNS, SS and SNS refer to the scores in the semantic and non-semantic conditions for blind and sighted participants respectively. The upper panel shows the percentages of single sounds correctly recalled. The middle panel shows the percentages of sequences correctly recalled. The lower panel shows the mean spans. Panels highlight that in the pre-test all groups perform similary, regardless of the specific condition. In the post-test instead, sighted participants perform singificantly better than blind individuals as indicated by the asterisks as indicated by the asterisks (one asterisk (*) represents p < 0.05, two asterisks (**) represent p < 001 and three asterisks (***) represent p < 0.001).

Finally, in order to test whether blind subjects could have had reached a performance at ceiling level already in the pre-test phase, we also analysed performance to the pre- and post-test phases (Fig. 4). The three parameters mentioned above were analysed by performing three different ANOVAs (one for each parameter as for the improvement analysis). Specifically, we ran 3-ways (3 × 2) mixed measures ANOVAs with *group* as between factor, *condition* and *phase* (pre- and post- test phases) as within factors. The analysis of variance revealed, for all three parameters, a significant interaction *group* × *condition* × *phase* for single sounds (F(1,20) = 17.99, p < 0.001) correct sequences (F(1,20) = 14.46, p < 0.01) and span (F(1,20) = 14.45, p < 0.01). Also in this case, post-hoc analysis were carried out with two tailed Student’s tests (both paired and unpaired) and Bonferroni correction was used in order to correct for multiple comparisons (p < 0.05 was considered significant). A total of 12 comparisons were conducted. With regard to the semantic condition (Fig. 4) our results showed that in the post-test, sighted participants recall a higher percentage of items compared to blind participants (unpaired t-test, the percentage of single sounds: t = 4.08, p < 0.01; percentage of correct sequences: t = 3.63, p < 0.05). Moreover, within group analysis shows that sighted participants perform better in the post-test phase compared to the pre-test phase in the semantic condition (paired t-test, the percentage of single sounds correctly recalled: t = 6.09, p < 0.01; percentage sequences correctly recalled: t = 4.13, p < 0.05). Comparison across conditions in sighted participants shows that they perform better in the semantic rather than in the non-semantic post-test (paired t-test, the percentage of single sounds: t = 6.05, p < 0.01; percentage of sequences correctly recalled: t = 4.37, p < 0.05). Regarding the span, the last of the three analysis, we observed a similar pattern of results. In the semantic condition, the span in the post-test phase was higher for sighted than blind participants (unpaired t-test: t = 3.37, p < 0.05). Furthermore, we observe a longer span for sighted individuals in the post-test phase compared to the pre-test phase in the semantic condition only (paired t-test: t = 4.21, p < 0.05). Finally, comparison across conditions reveal that sighted subjects reached a longer span in the semantic than non-semantic condition after the explorations, that is by comparing the span in the post-test phase across conditions (paired t-test: t = 3.90,p < 0.05). We did not find a significant difference between blind and sighted participants in the pre-test performance both in the semantic and non-semantic condition. Comparison between pre- and post-test shows that blind subjects performed in the same way in both conditions, they did not show a significant improvement after the exploration. In other words, regardless of the condition, the exploration did not influence the performance of the blind participants.

## Discussion

In the present study, our first aim was to understand the role of vision in the development of mental spatial images. In particular, we wanted to test whether a coherent semantic audio scene may improve performance in the proposed audio-spatial memory protocol and whether this effect would be affected by congenital blindness. The experiment included an exploration phase in which each participant explored the device, trying to build a mental image of the scene. We found that before the exploration both sighted and blind participants performed similarly regardless of the experimental conditions, either semantic or not. This result confirms that despite the lack of vision, blind individuals are still able to perform passive memory tasks. The Corsi test indeed, as shown by Vecchi and co-workers^11^, is a passive memory task, because the participant is just asked to remember sequences of spatialized sounds with no active manipulation of the stimuli. A passive task indeed mainly involves the storage of information. On the contrary, in an active task, participants have to transform, integrate or manipulate the information to be recalled^28^.

The difference between sighted and blind groups arises after the exploration in the semantic condition. The results indeed highlight that even if blind people can in fact create such mental image (both groups remember the same percentage of items’ positions), the capability in using such information is not as effective as in sighted individuals. One reason for this difference might rely on the fact that blind subjects seem to have difficulties in combining the spatial locations of the auditory items in a coherent and functional representation, even when they know stimulus positions in space. Although our task is passive by definition, the active manipulation of retained information in a coherent semantic scene might have been used in the post-test phase. With the introduction of coherence and meaning to the sound dispositions, sighted individuals were able to build, and later to use, a functional image of the scene that helped them in localizing and remembering the sounds. For this reason, it is likely that the significant difference observed for the improvements in the semantic condition between groups (see Fig. 2) derives from the higher ability showed by sighted participants in using the information gained during the exploration phase. As shown by De Beni and Cornoldi^25^, the lack of vision leads to specific difficulties when congenitally blind individuals have to create interactive images that involve several items at the same time. Simultaneous perception and manipulation of more than one object is typical of vision, while haptic and auditory perception, widely used by visually impaired individuals, mostly rely on sequential processing. Vision indeed facilitates simultaneous processing at high cognitive level^14^ thus the absence of vison might reduce the ability to properly process large amounts of information at the same time in visually impaired people. De Beni and Cornoldi^25^ have shown that congenitally blind individuals perform poorly in tasks involving a high spatial memory load. Our task involved 14 sounds spatialized in 25 positions which might have overloaded blind participants’ spatial memory. Thus, it can be suggested that visual experience plays an important role in providing the basis to deal with a functional representation of the scene, even if conveyed through the auditory modality.

However, the measure of improvement does not take into account the difference between groups in the pre- and post-test phases separately. Thus, it could be argued that the blind participants outperformed in both test phases, thus justifying their lower improvement. However, as shown in Fig. 4, we did not observe such a scenario. This aspect supports our conclusion that the improvement in sighted participants is most likely due to the exploration because, only this group of subjects shows a better performance in the post-test phase.

Furthermore, although semantic stimuli are generally easier to learn and to remember than non-semantic ones^37^, in the pre-test phase, semantic information does not improve the capability of memorizing the sequences. The improvement instead is present only after the exploration phase, supporting the hypothesis that only active manipulation of the retained information might meliorate memorization. In line with Mandler and colleagues^38^, storage of visual stimuli is enhanced when such stimuli are part of a scene coherently organized. In the pre-test phase, participants are not aware of the semantic coherence underlying stimuli positions in the context of the auditory scene. For this reason, before the exploration, the semantic information might not lead to better performance compared to the non-semantic condition. Thus, results highlight two main aspects. Firstly, semantic information per se does not aid memorization of the sequences. Participants focus more on the position of the sounds rather than their meaning. On the other hand, when the existence of a coherent scene composed by the stimuli to be memorized becomes clear via exploration, the semantic information leads to an improvement in memorization.

Finally, the strategy of memorization used by the two groups could have also played a role. Cornoldi and co-workers^39^ showed that the strategy used in accomplishing visual imagery tasks have implications on the patterns of the results. Sighted participants might have relied on the object-to-object (allocentric) relationship between stimuli in order to better recall the sequences in the post-test phase. As shown by Pasqualotto and colleagues^40^, sighted people generally use allocentric frames to represent spatial information while early vision loss may lead to an impairment in processing allocentric representations^41,42^. Along these lines, Thinus-Blanc and colleagues observed that early vision plays a fundamental role in establishing spatial relationships among the objects of the environment. Congenitally and early blind individuals indeed rely more on egocentric (centred to the body)^43^ rather than object-to-object or allocentric representations. As shown by Hollingworth^44^, in memory processes, the objects belonging to a memorized scene, are bond to the scene context. In other words, storage for a particular object is bound to the other elements of the scene. Moreover, the generation of mental images requires the use of both egocentric and allocentric representations^41,45^. In order to better remember sounds positions, sighted participants might have relied on the relations among the objects in the context of the audio scene. However, our test was not designed to differentiate the contribution of egocentric or allocentric frames in spatial memory, therefore future studies would be needed to further investigate this aspect.

In conclusion, our experimental paradigm allowed us to study how the exploration of a coherent audio semantic scene impacts on storing and recalling spatialized auditory stimuli. Although both blind and sighted individuals show similar audio spatial memory skills, the latter were the only ones able to take advantage of an active exploration of the semantic scene. These findings suggest that visual experience allows the simultaneous processing of multiple stimuli, even if presented through a non-visual modality (i.e. auditory). This result is also important in the context of developing novel solutions and technologies to enhance spatial representations in visually impaired individuals. On the other hand, blind individuals are still anchored to a sequential processing of information that does not allow them to take advantage of the semantic exploration of the scene. In order to test the validity of the presented paradigm, further studies might be pursued by extending properties of the spatialized auditory stimuli for instance by projecting them on a different plane than vertical. The development of extensions of the test presented here would provide a useful paradigm for cognitive evaluation in individuals whose spatial memory skills are rather difficult to test because of the absence of vision.

## Materials and Methods

### Participants

Twenty-two individuals participated in the study: 11 with visual disabilities (7 females, mean age ± SD: 41.82 ± 14.6070) and 11 without (7 females, mean age ± SD: 38.8182 ± 11.3033). The group of sighted participants reported no visual impairment and a visual acuity higher than 9/10. Information about blind subjects is presented in Table 1: all subjects are congenital blind according to international standards. Blind participants were recruited from the Italian Institute of Technology (IIT) database. The study was approved by the ethics committee of the local health service (Comitato Etico, ASL 3, Genova, Italy). All experiments were conducted in accordance with the Declaration of Helsinki (1964). Informed written consent was obtained for all the subjects. None of the participants had additional sensory disabilities or any kind of cognitive impairment. The test was presented in the form of a game which lasted for ~30 minutes.

**Table 1:** Clinical details of blind individuals.

Participants	Gender	Age	Pathology	Onset	Residual vision
A1	M	58	Glaucoma	Before Birth	No Vision
A2	M	57	Uvetis	Before Birth	Lights and Shadows
A3	M	51	Retinophaty	Before Birth	No Vision
A4	M	25	Leber’s Amaurosi	Since Birth	No Vision
A5	F	30	Retinitis pigmentosa	Since Birth	No Vision
A6	F	41	Glaucoma	Since Birth	No Vision
A7	F	52	Retinitis pigmentosa	Before Birth	Lights and Shadows
A8	F	27	Retinophaty	Before Birth	No Vision
A9	F	62	Atrophy eyball	Before Birth	No Vision
A10	F	30	Retinopathy	Since Birth	No Vision
A11	F	27	Microphthalmia	Since Birth	No Vision

### Stimuli

The audio test was carried out by means of a squared device (50 cm × 50 cm) composed of 25 blocks of 4 × 4 tactile sensors (each sensor measuring 2 cm × 2 cm) that register the position of each touch (Fig. 1). A single loudspeaker was positioned at the centre of each block of sensors to emit the sound stimulus, giving a total of 25 loudspeakers.

We used 26 sounds (13 semantic and 13 non-semantic) defining the two experimental conditions. Each sound stimulus lasted 3 seconds. Given that there were fewer than 25 sound stimuli, some loudspeakers reproduced the same sound. Regarding non-semantic stimuli, we used pure tones varying in frequency from 250 to 1300 Hz with a step size of 50 Hz; presentation order was pseudo-randomized across trials. To facilitate sound localization, we added a white noise sound to the original pure tone. Auditory stimuli, feedback sounds and the experimental procedure were implemented in Matlab (R2013a, The MathWorks, USA). Semantic sound stimuli were everyday sounds (e.g. thunderstorm, flying plane, the chirping of birds) downloaded from a royalty-free images web archive (Freesound.org). Each semantic sound was assigned to a specific loudspeaker in order to represent the scene of a day in the countryside (Fig. 1). Furthermore, we preserved a correspondence between semantic and non-semantic sounds. In other words, since some loudspeakers reproduced the same sound in the semantic condition, the same loudspeakers reproduced equal frequency sounds in the non-semantic condition.

### Experimental procedure

Subjects sat on a comfortable chair and the device was placed at a distance of ~30 cm from them, aligned to their body midline. Furthermore, while listening to the sounds, participants had to put their index finger on a plastic bottle lid fixed on the desk, which worked as the reference position. Participants were not allowed to see the device (sighted subjects were blindfolded before entering the room) but they could familiarize with it by exploring it with both hands before the beginning of the experiment. They were administered with a practice session to familiarize with the task in which only non-semantic sounds were used. The experiment consisted of a pre- and post- test phase, separated by an exploration phase. In the pre and post-test phases, subjects listened to sequences of sounds and were asked to spatially replicate the stimuli presentation order by touching the location from which the sound was emitted. Auditory feedback indicating response acquisition was provided to allow the participant to release her/his finger from the touched position. In order to make the test amusing for the subject, we employed a cat sound. After each trial, participants were asked to once again place the index finger on the starting position and wait for the next trial to start. The interval between two sequences of the same length was 1 s. The change in sequence length was verbally communicated by the experimenter. As in the original *Corsi* paradigm, we varied the length of the sequences during the test to be either two, three or four sounds. More specifically, we used 2 different sequences per length, for a total of 6 trials per block. However, the length did not increase based on participant performances as in the *Corsi* test, but each subject was tested with all the 6 sequences. The minimum distance between two consecutive sounds was set at 20 cm in order to make them easily discriminable.

Our aim was to test subjects’ performances before and after the exploration phase with sequences of different lengths. As the length of the sequences increases, the need for memory and spatial skills also increases. In the semantic condition, before starting the pre-test phase, participants heard the sounds one by one, and were asked to identify each of them. In the exploration phase, subjects were asked to freely touch the device surface while listening to sounds from the speakers activated after each touch without the help of the experimenter. They had five minutes to explore the haptic blocks and were instructed to touch the device with the index finger of their dominant hand. Each speaker was activated as soon the index finger touched a tactile sensor belonging to the haptic block. There was no pre-defined order for the exploration of each haptic block and the experimenter did not guide participants during this phase. In both conditions, participants were instructed to pay attention to the position of the sounds. In addition, at the beginning of the semantic exploration, subjects were asked to try to understand the audio scene. The experimenter told the participants that the semantic sounds were not randomly displaced on the surface but occupied specific locations to compose a meaningful scene (see Fig. 1). In addition, at the beginning of the semantic exploration phase, the experimenter instructed participants to try to image and remember the scenario created with the combination of the sounds. At the end of the post-test phase, subjects were asked to rebuild the auditory scene by locating each item presented in the semantic scene. In each trial, the experimenter read the name of one item and the participant was asked to locate the item by touching the device with the index finger of the dominant hand. Before releasing the finger, they had to wait for the feedback sound, reproduced by the central loudspeaker. They then replaced their finger on the reference position and waited for the next item.

For both modalities, performances were evaluated considering the percentage of correct sequences out of the total (6 sequences), the memory span and the percentage of single sounds correctly recalled out of the total (18 sounds). The span was calculated taking into account a validated procedure^46–48^. For all sequence lengths, the product of the number of correctly recalled trials and the sequence lengths was summed up and divided by 2 (i.e. the number of trials per sequence length), using the following formula:

1 $$\sum _{i=1}^{4}\frac{(\#corr.subtrials(i)\ast i)}{2}$$

Where *i* is the sequence length. According to (1), the span is between 1 and 10. We started with a sequence of 2 sounds, assuming that all participants were able to solve trials with a lower sequence length correctly. Thus, for the span we counted the trial of one sound as correct. For example, consider a subject who performs well for the sequence lengths of two and three items, but in the sequence of four audio items he performs only one trial correctly. In addition, the sequence of one was assumed correctly. Thus, this participant would have a span of $(1\ast 2+2\ast 2+3\ast 2+4\ast 1)/2=8$.

### Statistical analysis

In order to verify improvement after the exploration phase, we ran a 2-way (2 × 2) mixed-measures model ANOVA with the *group* (either blind vs. sighted) participants, as a between factor and *condition*, either (semantic vs. non- semantic), as a within factor. After this first analysis, we analyzed the scores in the pre- and post-exploration phases separately. More specifically, we performed a three-way (3 × 2) mixed-measures model ANOVA with the *group* as between factor and *condition* and *phase* (pre- and post- exploration phases) as within factors. In both cases, post-hoc analyses were carried out with two-tailed Student’s tests, both paired and unpaired. Bonferroni correction was used to test the significance of multiple comparison post hoc tests (p < 0.05 was considered significant).
